# Understanding Bone Metabolism Biomarker Variability Across the Menstrual Cycle: A Systematic Review

**DOI:** 10.1007/s00223-026-01482-1

**Published:** 2026-02-09

**Authors:** Isabel Guisado-Cuadrado, Nuria Romero-Parra, Paula Recacha-Ponce, Ana B. Peinado

**Affiliations:** 1 LFE Research Group, Department of Health and Human Performance, Faculty of Physical Activity and Sport Science-INEF, Universidad Politécnica de Madrid, Calle de Martín Fierro, 7, 28040 Madrid, Spain; 2 Department of Physical Therapy, Occupational Therapy, Rehabilitation and Physical Medicine, Faculty of Health Sciences, Universidad Rey Juan Carlos, Madrid, Spain; 3 Faculty of Health Sciences, Jaime I University, 12071 Castello de la Plana, Spain

## Abstract

Bone metabolism is a dynamic process regulated by systemic and local factors, including sex hormones. While in vitro studies suggest that hormonal fluctuations impact bone biomarkers, in vivo findings remain inconsistent. Therefore, this systematic review aimed to evaluate whether biochemical markers of bone resorption and bone formation fluctuate across different phases of the menstrual cycle in healthy regular menstruating females. Following PRISMA guidelines, a comprehensive search was conducted in PubMed and Web of Science (January 2025). A total of 18 studies were included and assessed for quality using the NHLBI Quality Assessment Tool. Considerable heterogeneity was found across studies in design, phase classification, and outcome reporting. While 5 out of 7 studies on β-CTX-I reported lower concentrations during the luteal phase, 6 out of 8 studies showed that osteocalcin concentrations did not vary across the MC. Findings concerning other biomarkers included in this review were limited and inconsistent, hindering the identification of a clear trend. Current evidence does not establish a consistent pattern in bone metabolism biomarker fluctuations across the MC. Variability in methodology, hormone verification, and participant characteristics may contribute to these inconsistencies.

*PROSPERO registration* CRD42022375821.

**Supplementary Information:**

The online version contains supplementary material available at 10.1007/s00223-026-01482-1.

## Introduction

Bone metabolism is a dynamic physiological process involving the continuous remodelling of bone tissue through the balanced activities of osteoblasts, which form bone, and osteoclasts, which resorb bone [1]. This balance is crucial for the maintenance of skeletal integrity and is influenced by multiple systemic and local factors, including hormonal fluctuations [2]. Among the key regulators of bone remodelling, sex hormones, such as oestrogens and progesterone, play an important role in modulating both osteoclast and osteoblast activity [3].

Despite this well-established relationship between ovarian sex hormones and bone metabolism, the extent to which menstrual cycle (MC) phases influence bone resorption and formation markers remains unclear. Several in vitro studies have demonstrated that oestradiol inhibits osteoclastogenesis by downregulating receptor activator of nuclear factor kappa-Β ligand (RANKL) and upregulating osteoprotegerin (OPG) expression, while also stimulating osteoblast activity via the Wnt/β-catenin pathway [4, 5]. In addition, in vitro studies on cultured human osteoblasts, suggest an osteoanabolic function of progesterone after 7 and 21 days of physiological progesterone exposure [6]. However, when these mechanisms are studied in vivo, the findings become more inconsistent. Some studies report significant MC-related variations in bone turnover markers [7–9], whereas others fail to detect meaningful differences [10, 11].

The physiological MC is characterized by rhythmic hormonal fluctuations, primarily driven by variations in oestradiol and progesterone levels, which regulate ovulation and menstruation while also exerting systemic effects on various physiological processes, including bone metabolism [12]. In eumenorrheic females, these cyclical hormonal changes provide a unique opportunity to explore their impact on biochemical markers of bone metabolism across different MC phases. Oestradiol levels rise gradually during the follicular phase, peaking just before ovulation, and reaching moderately elevated levels at the mid luteal phase. Progesterone, in contrast, exhibits a pronounced increase post-ovulation, reaching the highest concentrations during the mid luteal phase [13]. These hormonal changes are hypothesized to be a source of variability [14, 15], indicating that reduced bone resorption—measured by beta-C-terminal telopeptide of type I collagen (β-CTX-I)—during the luteal phase, suggesting that hormonal fluctuations may influence the activity of bone remodelling markers. This implies that the MC phase constitutes a critical variable that should be standardised to ensure accurate interpretation of bone metabolism outcomes. Therefore, this study aimed to systematically analyse existing literature in which the variations in bone metabolism markers across the different phases of the MC were examined. Specifically, it seeks to determine whether bone resorption markers – e.g. β-CTX-I, parathyroid hormone (PTH), sclerostin, RANKL, tartrate-resistant acid phosphatase 5b (TRACP5b) – and bone formation markers – e.g., PINP, bone-specific alkaline phosphatase (BALP), osteocalcin, OPG, and calcitonin – exhibit significant phase-dependent fluctuations.

## Methods

This systematic review was conducted following PRISMA guidelines (for Prisma checklist see Supplementary Material 1). The review protocol was registered in PROSPERO in 2022 (CRD42022375821). A comprehensive systematic search was initially performed in PubMed and Web of Science in 2022 and subsequently updated on January 8, 2025, to identify relevant studies.

### Study Inclusion and Exclusion Criteria

Participants from eligible studies met the following criteria: (a) aged 18 to 45 years; (b) premenopausal naturally menstruating females; (c) were not using hormonal contraceptives or medications that could influence the hypothalamic-pituitary-ovarian axis (HPO); (d) had no MC-related disorders (e.g., amenorrhea, polycystic ovary, etc.) or other conditions known to disrupt the HPO axis; and (e) had no pathologies related to bone metabolism. Studies were included in the analysis if they met the following criteria: (a) aimed to evaluate changes in bone metabolic marker concentrations (β-CTX-I, PTH, sclerostin, RANKL, TRACP5b, PINP, BALP, osteocalcin, OPG, and calcitonin), (b) performed within-group comparisons, and (c) measured outcomes during two or more clearly defined phases of the MC. No restrictions were applied regarding the date of publication.

### Search Strategy for Identification of Studies

A systematic electronic literature search was conducted by IGC to identify all relevant articles using two online databases (PubMed and Web of Science). The search strategy incorporated terms related to the MC and bone metabolism. For the MC, terms included “menstrual cycle” OR “menstrual phase” OR “follicular phase” OR “luteal phase”. For bone metabolism, terms included “bone remodelling” OR “bone (re)modelling” OR “bone metaboli*” OR “bone resorption” OR “bone formation” OR “bone markers” OR “bone turnover”. Studies involving non-relevant populations or conditions were excluded by applying terms such as “postmenopausal” OR “transgender” OR “cancer” OR “syndrome” OR “endometriosis” OR “mice” OR “rats” OR “animal” (See database search details in Supplementary Material 2).

### Data Selection, Extraction and Study Quality Assessment

#### Selection of Studies

Two reviewers (IGC and ABP) independently screened the article titles and abstracts to identify studies for eligibility, removing duplicates using EndNote. In the initial phase, the eligibility of each title and abstract retrieved was evaluated against the pre-established inclusion and exclusion criteria. Studies that clearly failed to meet the inclusion criteria or matched any exclusion criterion were removed during this stage. In the second phase, full texts were reviewed and further assessed based on the predetermined criteria. Any disagreements between the reviewers regarding study eligibility were resolved by consulting a third reviewer (NRP).

#### Data Extraction and Management

Data extraction was performed by IGC using a pre-designed data extraction form, and the results were independently verified by ABP and NRP. Any disagreements were addressed by re-evaluating the original article, and consensus was reached through discussion during review meetings involving all four team members (IGC, NRP, PRP and ABP). When available, percentage mean differences (%MD) between MC phases were extracted directly as reported in the original studies. When %MD values were not reported, they were calculated from phase-specific mean concentrations as:

$$\:(B-A)/A\times\:100$$

where *A* represents the first MC phase, considering day 1 as the first day of menstrual bleeding, and *B* represents the subsequent phase along this timeline. When numerical data were reported only in graphical format, values were extracted using WebPlotDigitizer (version 4.8).

### Standardization of the MC Phases

To standardize the terminology related to the MC phases, the following criteria have been followed: the early follicular phase (EFP) indicated by the onset of bleeding until day 5, corresponding to phase 1 of Elliott-Sale, Minahan, De Jonge, Ackerman, Sipilä, Constantini, Lebrun and Hackney [13]; the mid follicular phase (MFP) is positioned between the EFP and the late follicular phase (LFP); the LFP occurs in the 14–26 h prior to ovulation and the LH surge, phase 2 of Elliott-Sale et al. [13]; the ovulatory phase is indicated by a positive urinary ovulation kit and lasts 24–36 h, phase 3 of Elliott-Sale et al. [13], or alternatively, by the detection of the LH peak in blood if daily measurements have been taken; the early luteal phase (ELP) extends from the ovulatory phase to the mid luteal phase (MLP); the MLP occurs + 7 ± 1 days after ovulation has been confirmed, phase 4 [13, 16]; and the late luteal phase (LLP) extends from the MLP until the next menstrual bleeding (See Table 2 for a summary of the phases included in each study).

**Table 1:** Characteristics of the selected studies.

References	Sample size (*n*)	Age	Timing	Fed or fasted	Resting time	Sample type
Buchanan et al. [25]	20	24–44	9 a.m.	Overnight fasting	Not reported	Serum
Chiu et al. [20]	20	34 (31–36)	7–10 a.m.	Overnight fasting	Not reported	Serum
Gass et al. [7]	55	27.3 ± 4.81	8–10 a.m.	Overnight fasting	Not reported	Serum
Gorai et al. [22]	10	33 ± 7	8–9 a.m.	Overnight fasting	Not reported	Serum and urine
Guisado-Cuadrado et al. [17]	8	30.45 ± 5.28	8–10 a.m.	Fed 2 h prior	24 h	Serum
Guisado-Cuadrado et al. [9]	9	25.5 ± 4.1	8–11 a.m.	Fed 2 h prior	24 h	Serum
Guzman et al. [11]	10	22 ± 3	9 a.m.	Fed 30 min prior	12 h	Plasma
Iida et al. [23]	42	19.6 ± 0.8	7–9 a.m.	Not specified	Not reported	Serum and urine
Jürimäe et al. [26]	24	18.3 ± 1.6	Morning	Overnight fasting	Rested	Plasma
Lee et al. [18]	14	19.9 ± 0.8	Morning	Overnight fasting	Not reported	Serum
Martin et al. [10]	14	21 ± 2	08 a.m. ± 30 min	Overnight fasting	Rested state (24 h)	Serum
Massafra et al. [24]	12	24.2	8–9 a.m.	Overnight fasting	Not reported	Serum
Mozzanega et al. [8]	20	24.3 ± 3.6	Not reported	Not specified	Not reported	Serum
Nielsen et al. [21]	8	33	8 a.m.	Overnight fasting	Not reported	Serum
Pitkin et al. [19]	7	22–31	8 a.m.	Overnight fasting	Not reported	Plasma
Schlemmer et al. [28]	10	33	8 a.m.	Overnight fasting	Not reported	Serum
Shimizu et al. [27]	15	21.5 ± 1.1	Afternoon (14: 00)	Fed 1 h prior	Not reported	Serum
Zittermann et al. [29]	10	25.1 ± 3.0	7 a.m.	Overnight fast (12 h)	Not reported	Serum

*Timing* refers to the time of day when blood or urine samples were collected. *Fed or fasted* indicates the nutritional state of participants prior to sampling (e.g., overnight fasting, fed a certain number of hours before). *Resting time* refers to the time participants remained at rest prior to sample collection. *Sample type* specifies the biological sample used for biomarker analysis

**Table 2:** MC phases in which bone metabolism marker concentrations are provided in each study.

	Early follicular	Mid follicular	Late follicular	Ovulatory	Early luteal	Mid luteal	Late luteal
Buchanan et al. [25]^a^	✓		✓		✓		✓
Chiu et al. [20]^b^	✓	✓	✓	✓	✓	✓	✓
Gass et al. [7]^b^	✓	✓	✓	✓	✓	✓	✓
Gorai et al. (1995)^b^	✓	✓	✓		✓	✓	✓
Guisado-Cuadrado et al. [17]	4 ± 1		12 ± 2			21 ± 3	
Guisado-Cuadrado et al. [9]	4 ± 1		12 ± 3			23 ± 2	
Guzman et al. [11]		8 ± 1				22 ± 3	
Iida et al. [23]	✓			✓			
Jürimäe et al. [26]		8 ± 3				20 ± 2	
Lee et al. [18]	Days: 1 and 3–5			16.6 ± 2.8			29.3 ± 3.9
Martin et al. [10]	Days 2–3			Day after + LH		7–8 days after + LH	
Massafra et al. [24]^b^	✓	✓	✓		✓	✓	✓
Mozzanega et al. [8]	Days 2–4		Days 12–14				Days 24–26
Nielsen et al. [21]^b^	Day 2	✓	✓	✓	✓	✓	Day before menstruation
Pitkin et al. [19]^b^	✓	✓	✓	✓	✓	✓	✓
Schlemmer et al. [28]^b^	✓	✓	✓	✓	✓	✓	✓
Shimizu et al. [27]		8.4 ± 3.9				23.8 ± 5.1	
Zittermann et al. [29]	Day 3		3 days before + LH		3 days after + LH	✓	3 days before bleeding

^a^Divide the MC into weeks. ^b^Blood samples are collected on alternate days. ✓ measurements were taken during that phase, but the exact day or range of days cannot be specified

### Quality Assessment of Included Studies (Risk of Bias)

**Fig. 1:**
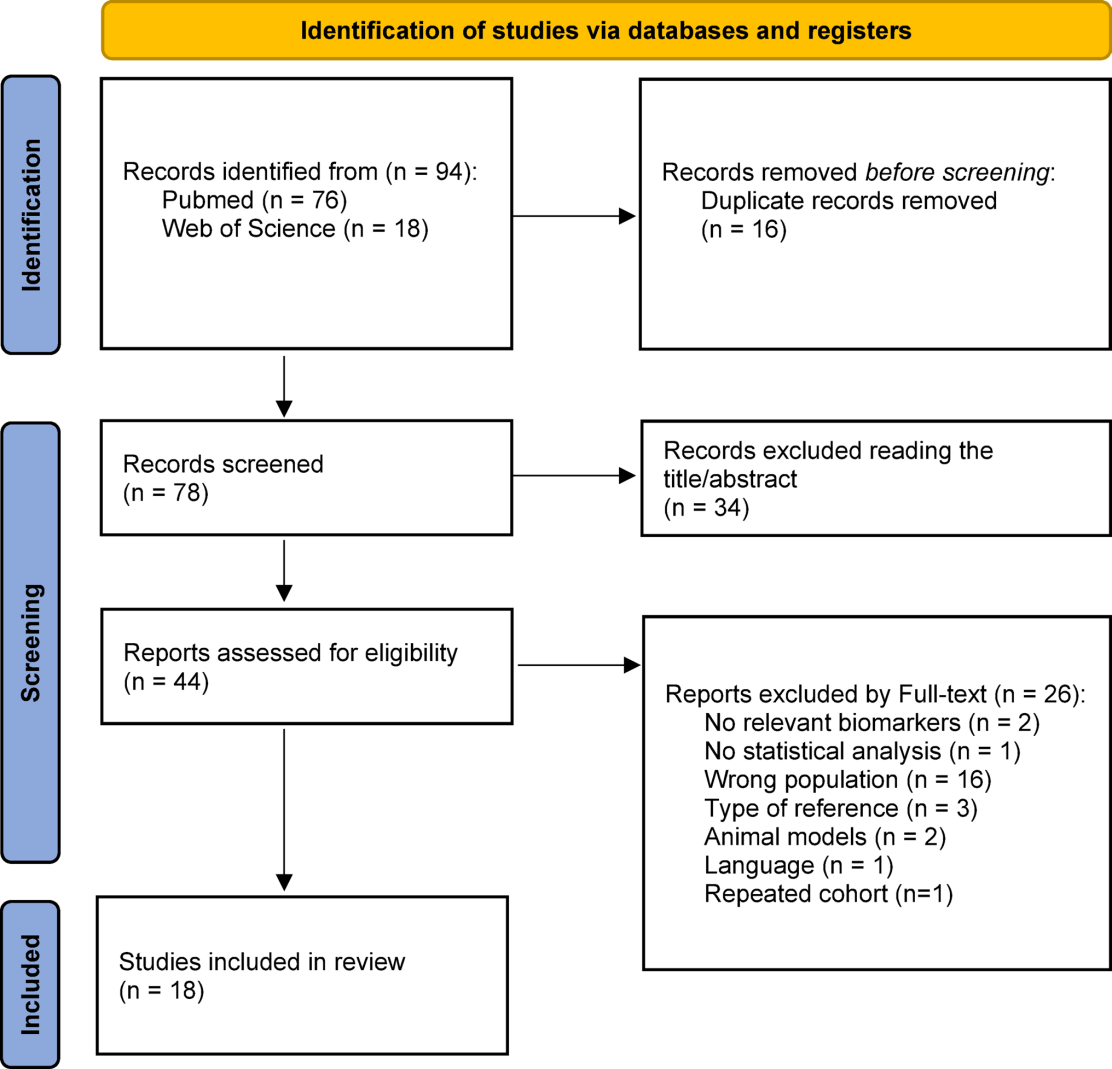
Summary of the quality assessment results using the national heart, lung, and blood institute quality assessment tool for observational cohort and cross-sectional studies, highlighting the areas with a high risk of bias

Eighteen studies were evaluated for quality using the National Heart, Lung, and Blood Institute Quality Assessment Tool (NHLBI-QAT) for Observational Cohort and Cross-Sectional Studies. This tool includes 14 items (See items in Fig. 1) aimed at assessing the internal validity of observational and cross-sectional research. Each item is rated as ‘yes’ (indicating low risk of bias), ‘no’ (high risk of bias), or ‘not reported’ (unclear risk of bias). Additionally, given the low quality of the existing evidence related to the MC [16], a qualitative evaluation of the methodology used to verify and determine the MC phase in each study was conducted.

## Results

### Study Selection and Study Selected Characteristics

**Fig. 2:**
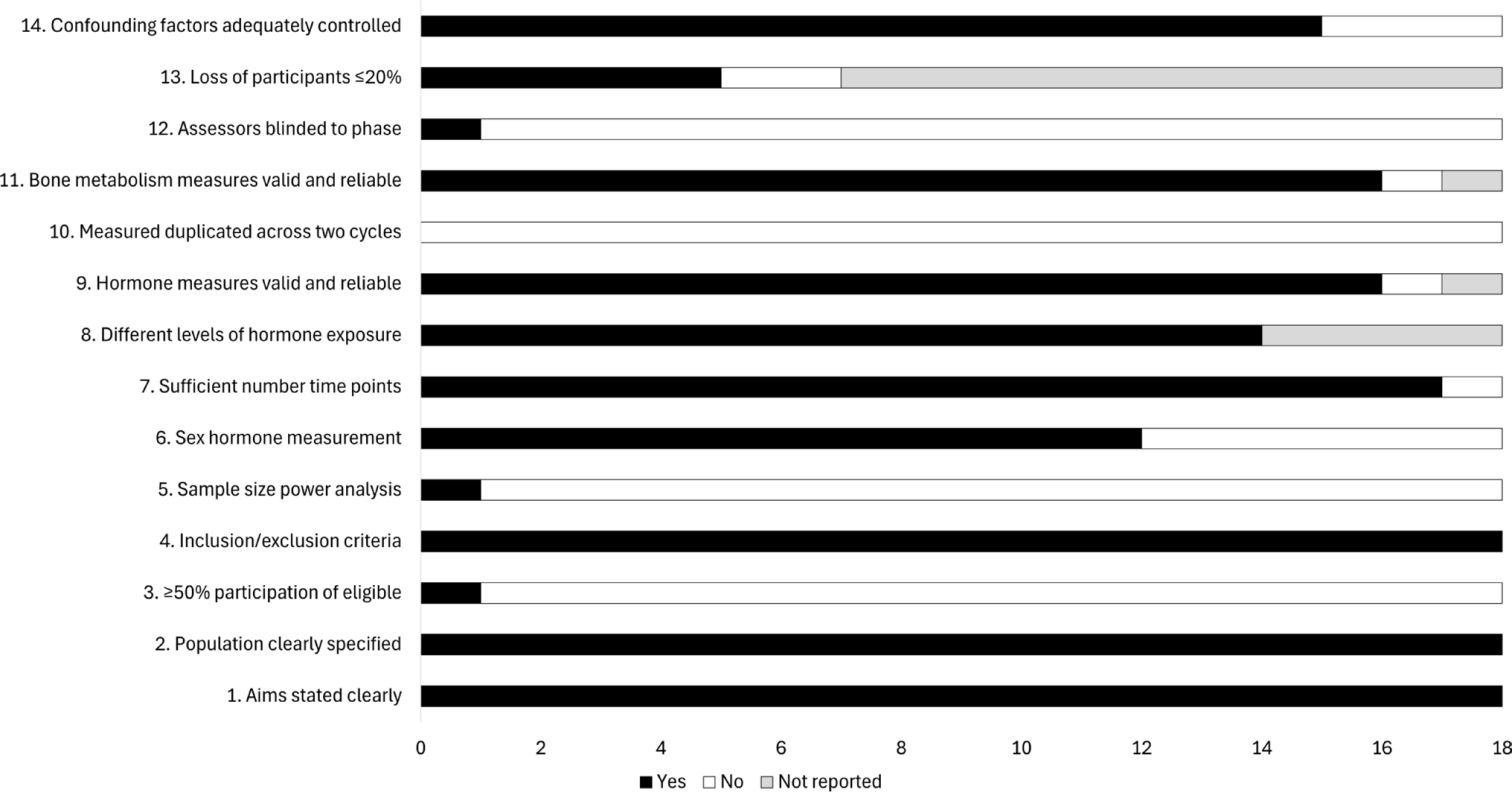
Flow diagram showing the study selection process used in this review article (*n* = 18)

The initial database search identified 94 articles. After removing duplicates, 78 records were screened for eligibility based on their title and abstract. Following this screening process, 34 articles were excluded based on their title and/or abstract, leaving 44 articles for full-text assessment. Among these 44 articles, 26 were excluded based on the reasons shown in the Fig. 2. Finally, a total of 18 studies included in this qualitative analysis. The main characteristics of the included studies are presented in Table 1. Due to the high heterogeneity in study designs and outcomes, as well as the limited number of studies evaluating the same biomarker, a quantitative analysis was not deemed appropriate. Therefore, the findings are presented in a narrative synthesis.

### Methodological Quality

#### Quality Assessment of Included Studies

Figure 1 presents an overview of the NHLBI-QAT results, highlighting the study design features with the highest risk of bias. Study scores ranged from 5 to 10 out of a possible 14 points (see Supplementary Material 3), with a mean score of 8.4 ± 1.2.

#### MC Phase Identification and Verification

Of the 18 included studies, 6 of them detected peak LH with urine detection kits [9–11, 17–19], 4 studies determined peak LH by successive LH blood tests [7, 20–22], 1 study detected ovulation by ultrasound [8], 1 study by the observation of fernlike crystals [23], 1 study performed measurements on successive days to detect pre-ovulatory peak oestradiol [24] and 5 studies did not use any method to directly detect ovulation [25–29]. To verify the phases of the MC with sex hormone concentrations in blood, 12 studies reported oestradiol and progesterone concentrations corresponding to the phases of the MC under study [7, 9, 11, 17, 18, 20–22, 24–26, 29], 3 studies only reported oestradiol concentrations [10, 23, 27], 1 study reported oestradiol concentrations for some participants only (not the whole sample) [19], 1 study did not report hormone concentrations although the authors claim that the hormone pattern is correct [8], and 1 study reported individual data of each participant of oestradiol and progesterone across the MC [28]. Table 2 shows MC phases which were measured in each study.

### Bone Metabolism Outcomes

**Fig. 3:**
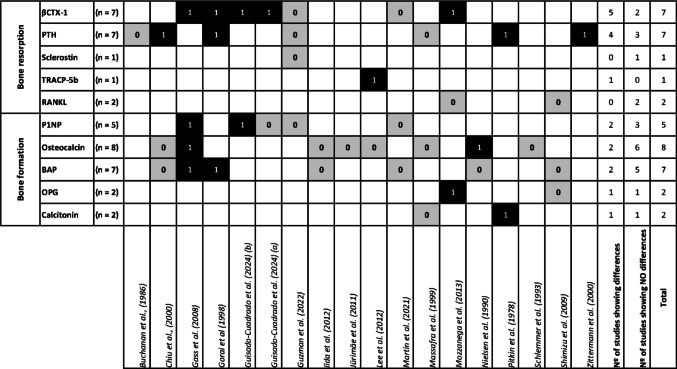
Variations in bone metabolism markers across the MC. Black cells (1) indicate studies that found significant differences between phases, while grey (0) represent studies that found no differences. White cells indicate that the study did not analyse that specific marker. The last two columns summarize the number of studies reporting differences or no differences for each marker

**Fig. 4:**
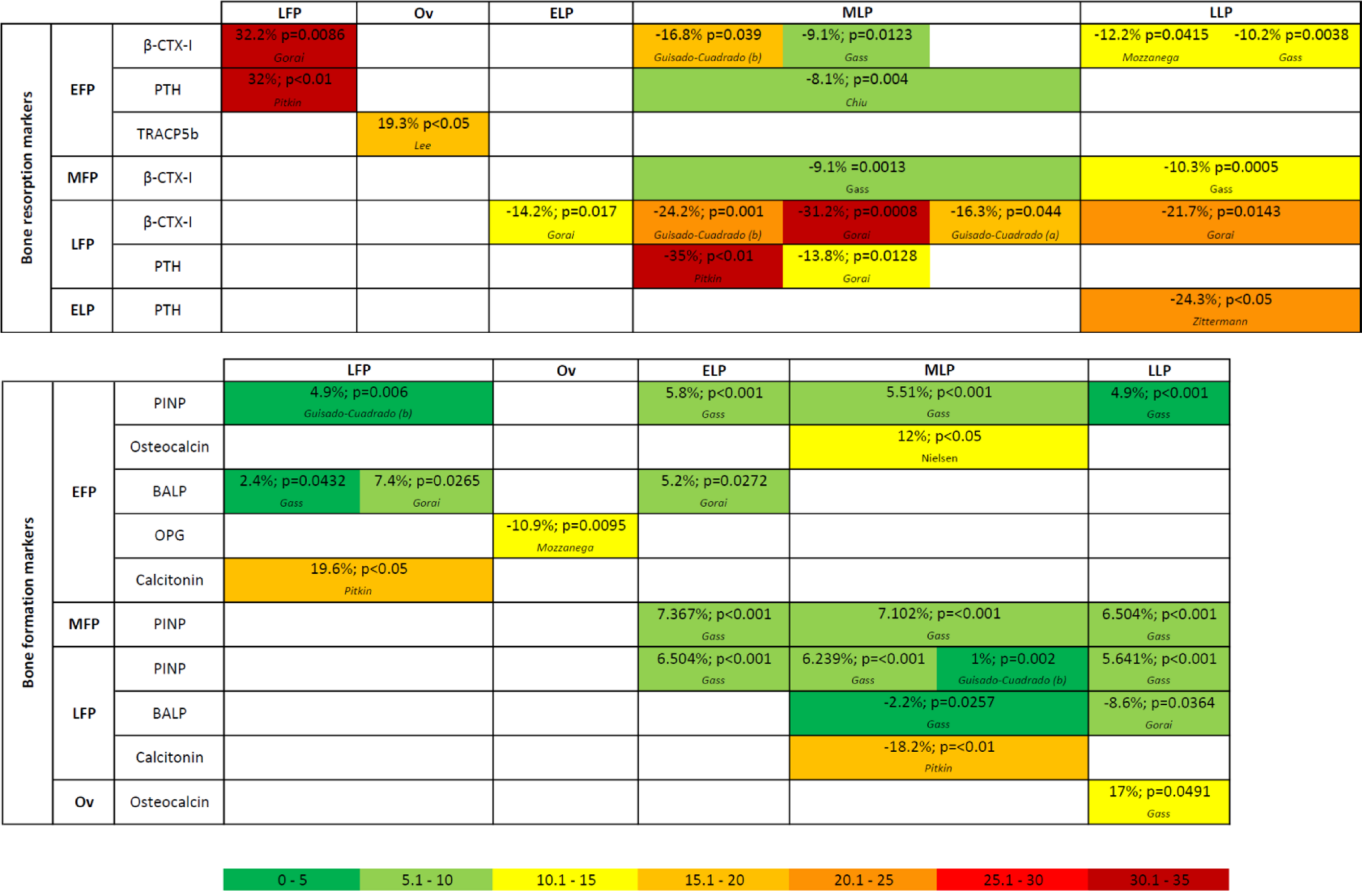
Heatmap of within-study percentage differences (%Δ) in bone resorption (upper panel) and bone formation (lower panel) markers across menstrual cycle phases. %Δ represents the difference in mean concentrations between the reference phase (row) and the comparison phase (column). Cell colours reflect the magnitude of the percentage difference according to the colour scale shown (from 0–5% to > 30%). EFP, early follicular phase; MFP, mid follicular phase; LFP, late follicular phase; Ov, ovulatory phase; ELP, early luteal phase; MLP, mid luteal phase; LLP, late luteal phase

An overview of the main significant differences between MC phases in bone resorption and formation markers is shown in Fig. 3. When studies reported significant differences between phases, quantitative information on the magnitude and direction of these changes was summarized using within-study percentage differences between phases, as illustrated in Fig. 4. Only statistically significant comparisons reported in the original studies are shown.

#### Bone Resorption Markers

##### β-CTX-I

Seven studies analysed the effect of the MC on β-CTX-I. Guzman et al. [11] and Martin et al. [10] found no significant effect of the MC phase. In contrast, 5 studies [7–9, 17, 22] identified significant variations across phases. Gass et al. [7] showed higher concentration in the EFP and MFP compared to MLP and LLP; Gorai et al. [22] showed higher concentrations in the LFP compared to EFP, ELP, MLP, and LLP; Guisado-Cuadrado [9] observed lower concentrations in the MLP compared to the LFP and EFP; Guisado-Cuadrado [17] reported lower concentrations in the MLP compared to the LFP and; Mozzanega et al. [8] reported higher concentrations in the EFP than in the LLP.

##### PTH

Seven studies analysed PTH fluctuations throughout the MC revealed mixed findings. Three studies [11, 24, 25] found no significant differences in PTH levels across menstrual phases. In contrast, four studies reported significant variations: Chiu et al. [20] observed higher PTH concentrations in the follicular phase (mean values of EFP, MFP and LFP) compared to the luteal phase (mean values of ELP, MLP and LLP), Gorai et al. [22] showed higher concentrations in the LFP compared to the MLP, Pitkin et al. [19] found higher PTH levels in the LFP compared to both the EFP and MLP, and Zittermann et al. [29] reported higher PTH concentrations in the ELP compared to the LLP.

##### Sclerostin

Only Guzman et al. [11] has reported sclerostin concentrations across the phases of the MC, showing no effect for phase (MFP vs. MLP).

##### TRACP5b

Lee et al. [18] showed that TRACP5b concentrations were higher in the ovulatory phase than in the EFP.

##### RANKL

Two studies investigated the changes in RANKL concentrations across the MC [8, 27]. These investigations showed no variation was observed in RANKL values at any time.

#### Bone Formation Markers

##### PINP

Five studies examined the variation of PINP across the phases of the MC. Guisado-Cuadrado [9], Guzman et al. [11], and Martin et al. [10] showed that there were no differences between MC phases. While Gass et al. Gass, Kagan, Kohles and Martens [7] observed significantly lower concentrations in the EFP, MFP and LFP compared to the ELP, MLP and LLP; and Guisado-Cuadrado et al. [17] showed higher concentrations in the LFP compared to the MLP and EFP.

##### Osteocalcin

Eight studies compared osteocalcin concentrations across the phases of the MC. Six studies [10, 20, 23, 24, 26, 28] reported no significant menstrual phase-dependent variations in osteocalcin concentrations. In contrast, Nielsen et al. [21] reported that osteocalcin concentrations were lower in the EFP compared to the MLP and Gass et al. [7] showed lower concentrations in the ovulatory phase compared to the LLP.

##### BALP

Seven studies examined the variation of BALP across the phases of the MC. Five of these studies [10, 20, 21, 23, 27] reported no differences in BALP concentrations across MC phases. Gass et al. [7] reported higher concentrations in the LFP compared to EFP and MLP; and Gorai et al. [22] showed lower concentrations in the EFP compared to the LFP and ELP, and lower in the LLP compared to the LFP and ELP.

##### OPG

Two studies have compared OPG concentrations across different phases of the MC [8, 27]. Shimizu et al. [27] showed no changes in OPG concentrations between MC phases, while Mozzanega et al. [8] showed higher concentrations in the EFP than in the ovulatory phase.

##### Calcitonin

Two studies showed calcitonin concentrations across the MC. Massafra et al. [24] showed no significant cycle-dependent variations in calcitonin concentrations. Pitkin et al. [19] showed higher calcitonin concentrations during the LFP compared to the EFP and MLP.

## Discussion

### MC Phase Determination and Verification

Accurate identification of MC phases is crucial when investigating the effects of hormonal fluctuations on bone metabolism. The studies included in this review employed different methods to determine ovulation and phase classification, which introduces methodological variability that may contribute to inconsistencies in findings. Among the 18 studies analysed, 5 studies did not use any direct method to confirm ovulation, which raises concerns about the accuracy of phase verification. In addition to ovulation detection, verifying MC phases through blood hormone concentrations (oestradiol and progesterone) enhances the reliability of phase classification. In this review, 12 studies reported both oestradiol and progesterone concentrations, which is the most robust approach to confirm menstrual phase status. However, 3 studies only reported oestradiol levels, one study provided oestradiol concentrations for a subset of participants, and one study did not report any hormone concentrations, relying solely on assumed hormonal patterns. The absence of direct hormonal verification in some studies may introduce misclassification bias, as inter-individual variability in oestradiol and progesterone levels can lead to discrepancies between calendar-based phase estimations and actual hormonal profiles. Moreover, aside from the inability to classify the phases accurately, non-eumenorrheic hormonal patterns may also be included (e.g., deficient luteal phases) [13]. The lack of uniformity in ovulation detection and phase verification across studies complicates direct comparisons of results. The timing of measurements within each phase is particularly relevant, as oestradiol levels increase progressively during the follicular phase and peak just before ovulation, making the late follicular phase highly variable depending on when it is measured. Similarly, if the early follicular phase is defined too late (e.g., beyond day 7–8), oestradiol levels may already be rising, and the phase may no longer represent a low-hormone baseline. This variation in phase classification could explain some of the inconsistencies in bone metabolism marker fluctuations across studies.

### Bone Resorption Markers

Bone resorption is primarily driven by osteoclast activity, which is influenced by regulatory factors such as PTH, RANKL, and sclerostin. Bone resorption can be assessed through intermediate markers such as β-CTX-I and TRACP5b, which reflect different aspects of osteoclastic activity, being β-CTX-I the recommended indicator by the International Osteoporosis Foundation (IOF) and the International Federation of Clinical Chemistry (IFCC) to standardized immunoassays in both observational and interventional studies. Regulatory factors play a direct role in modulating osteoclast differentiation, activation, and lifespan [30], while biochemical markers reflect the extent of bone resorption rather than actively regulating the process [15] (see Fig. 3 for an overview of the results from each study). The findings of this review suggest inconsistent results regarding bone resorption biomarkers across the MC. While some studies report phase-dependent variations, others do not observe significant differences between phases, reflecting the complexity and individual variability of bone metabolism in response to physiological hormonal fluctuations.

β-CTX-I is a well-established marker of bone resorption, reflecting osteoclastic activity and collagen degradation. In vitro studies show that oestradiol downregulates sclerostin, an osteocyte-secreted inhibitor of the Wnt/β-catenin pathway, which plays a critical role in maintaining osteoblast activity [4]. The results of this review show that 5 out of 7 studies found significant phase-dependent fluctuations in β-CTX-I concentrations, specifically, higher concentrations LFP compared to MLP/EFP/LLP. Given that oestradiol levels increase during the LFP and peak at ovulation, its inhibitory effect on osteoclast activity could explain the observed decrease in β-CTX-I levels during lower oestradiol concentration phases. Notably, when the magnitude of within-cycle changes is considered, bone resorption markers, including β-CTX-I, appear to exhibit greater phase-related variability than bone formation markers, as illustrated in Fig. 4. However, these MC-related changes remain modest when benchmarked against other major sources of biological variability. In particular, β-CTX-I displays pronounced circadian variation, with peak concentrations during the early morning hours and nadir levels around midday, resulting in fluctuations of approximately 40–66% across the day [15]. Furthermore, physiological and clinical factors such as recent exercise or bone fracture are associated with substantially larger changes in β-CTX-I concentrations, with post-fracture increases reaching up to 150%, underscoring that diurnal variation, physical stressors, and clinical conditions are likely to exert a greater influence on β-CTX-I interpretation than MC phase alone [15].

A key regulator of bone resorption is PTH playing a crucial role in calcium homeostasis and bone remodelling by increasing osteoclast activity. In vitro findings indicate that oestradiol directly suppresses PTH secretion from the parathyroid glands and that its decline leads to increased circulating PTH [31]. Additionally, oestradiol in vitro inhibits PTH-induced osteoclastogenesis via the cAMP-PKA pathway, reducing bone resorption [32] and it has been observed to enhance osteoblast function in vitro and counteracts PTH’s inhibitory effects on bone formation [33]. The present findings are mixed, with four studies reporting significant higher concentrations in phases when oestradiol is elevated [19, 22, 29] and three studies finding no differences. Given the inconsistency of current findings, it is plausible that sex hormone variations during the MC may constitute a secondary regulatory factor in bone metabolism, specifically in PTH regulation, whose effects may be overshadowed by other systemic or local modulators.

Another critical regulator of bone remodelling is sclerostin. While in vitro studies have showed clearly that oestradiol downregulates sclerostin expression by interacting with oestrogen receptor β (ERβ) and modulating the Wnt/β-catenin pathway – thereby promoting bone formation [4] – the present systematic review does not provide sufficient evidence to conclude that oestradiol fluctuations throughout MC are a strong enough stimulus to regulate this mechanism. Only one included study [11] investigated sclerostin level fluctuations across the MC and found no significant differences between phases. However, a single study is insufficient to draw definitive conclusions. Similarly, RANKL—another key regulator of bone remodelling that promotes osteoclastogenesis by binding to RANK on osteoclast precursors, thereby stimulating their differentiation and activation [2] was assessed in two studies included in this review, neither of which reported significant variations across the MC. Consistently, the findings suggest that oestradiol variations throughout the MC alone may not significantly regulate bone metabolism and should be viewed as one contributing factor within a multifactorial regulatory framework.

Finally, TRACP5b, an osteoclast-derived marker of bone resorption, was assessed in a single study [18], showing higher concentrations in the ovulatory phase compared to menstruation. Given that ovulation is characterized by a sharp increase in oestrogen followed by a progesterone surge, it is unclear whether TRACP5b fluctuations are oestrogen-mediated or reflect other metabolic shifts related to the ovulatory process.

### Bone Formation Markers

Bone formation is primarily mediated by osteoblast activity, which is influenced by oestradiol and other regulators, including such as hormonal factors, mechanical loading, nutritional intake, aging, energy availability, inflammation, etc. The biomarkers examined in this review include PINP, osteocalcin, BALP, OPG, and calcitonin (see Fig. 3 for an overview of the results from each study).

Serum PINP is a bone (re)modelling marker produced during the synthesis of type I collagen, which constitutes 90% of the bone matrix and is the most abundant bone protein [15]. Among the five studies analysed, three found significant variations, whereas three reported no differences. Of the two studies that found differences in PINP levels throughout the MC, the phase showing the highest PINP concentrations did not coincide. These inconsistencies point to the possibility that oestradiol fluctuations throughout the MC are not a primary determinant of bone formation. As previously noted, bone metabolism is regulated by multiple interacting factors, which may reduce the isolated impact of oestradiol variations during the MC. Moreover, pre-analytical and physiological factors (e.g. assay-specific characteristics, sample storage conditions, fasting status) and physical stressors, such as intensive exercise or bone fracture, are associated with changes in PINP concentrations that substantially exceed those reported across MC phases. Collectively, these observations reinforce the notion that MC-related effects on PINP are modest in magnitude and are unlikely to outweigh other sources of biological and pre-analytical variability when interpreting bone formation markers [15].

Serum osteocalcin is regulatory molecule recognized as a biomarker of osteoblast activity, with its levels indicating the rate of bone formation [34]. Osteocalcin is the most prevalent non-collagenous protein in bone and plays a role in matrix mineralization through its calcium-binding properties [34]. In vitro investigations showed that oestrogen-related receptor α (ERRα) interacts cooperatively with PGC-1α and directly regulates osteocalcin gene expression in vitro, suggesting that ERRα might exert their physiological roles in bone formation by modulating osteocalcin gene expression [35]. When examining whether the physiological fluctuations of oestradiol across the MC modulate this biomarker in vivo, the evidence is limited. Six out of eight studies included in this review reported no significant differences in osteocalcin concentrations between MC phases, suggesting that short-term physiological sex hormonal changes may not be a major modulator to influence bone formation markers at the systemic level.

In the same line, BALP is a bone-specific enzyme produced by osteoblasts that plays a key role in the mineralization process by increasing local phosphate availability and promoting hydroxyapatite crystal formation, thereby reflecting bone formation [36]. BALP concentrations remained stable across menstrual phases in most of the studies included, as it is shown by 5 studies out of 7.

To further explore bone formation dynamics, OPG, a decoy receptor for RANKL that inhibits osteoclast activity [3], was examined in two studies. In vitro evidence indicates that oestradiol upregulates OPG expression in stromal cells via oestrogen receptor-α (ERα), indicating a direct stimulatory effect of oestradiol on OPG production [5]. When assessing short-term changes in OPG across the MC, in vivo findings are limited and inconsistent: one study found no significant differences between phases, while another reported lower concentration in the EFP compared to the LFP. These results suggest that the physiological fluctuations of oestradiol throughout the MC may not be sufficient, as a primary factor, to consistently regulate OPG concentrations; however, the available evidence is very limited.

Finally, calcitonin’s primary function is to counteract PTH-induced bone resorption. In vitro studies have shown that oestradiol can stimulate calcitonin secretion by rapid, direct, and specific effects on the thyroid C cell, inhibiting bone resorption (Greenberg et al. 1986). This biomarker was analysed in two studies included in this review: one reported no significant differences across MC phases [24], while the other observed higher calcitonin levels in the LFP compared to the EFP ns MLP [19]. These divergent findings highlight the need for further research to clarify the potential influence of MC-related hormonal fluctuations on calcitonin secretion.

### Methodological Considerations

Overall, the findings of this review indicate that no clear pattern has been detected in bone metabolism markers across the MC; moreover, there is a high degree of variability in the reported results. This variability can be attributed to several methodological and physiological factors that should be carefully considered when interpreting MC-related changes in bone metabolism.

One of the primary challenges in studying bone metabolism across the MC is the high inter- and intra-individual variability in hormonal responses. While all participants included in these studies were classified as eumenorrheic, this classification does not account for differences in absolute oestradiol levels between individuals. It is possible for one subject to have lower oestradiol concentrations than another while still exhibiting a hormonal profile that aligns with the eumenorrheic criteria. This individual variability in hormonal exposure may contribute to discrepancies in findings across studies, as some individuals may experience greater fluctuations in bone metabolism markers in response to oestradiol than others.

Another important source of variability is the lack of uniformity in defining MC phases across studies. While many studies classified the EFP as the control phase due to its low concentrations of ovarian sex hormones, others measured at day 8 of the cycle, which would already correspond to the MFP when oestradiol levels are typically higher. This variation could contribute to differences in baseline values of bone metabolism biomarkers, making it more difficult to detect phase-related changes. Similarly, there are differences in the timing of measurement in the middle of the MC. Some studies measured before the LH surge, aiming to capture the oestradiol peak (LFP); others measured after the LH surge (ovulatory phase), when oestradiol concentrations start to decrease; while other studies measured even later, capturing the ELP. From a methodological standpoint, this approach is valid provided that the timing of measurements is clearly defined; however, it contributes to variability in the findings when interpreting results across studies.

Beyond MC variability, differences in the physical condition of study participants introduce another layer of heterogeneity. Bone metabolism is influenced by physical activity, and mechanical loading, all of which can vary widely among study participants. Individuals who engage in regular high-impact exercise or resistance training may exhibit higher baseline bone remodelling, potentially influencing how their bone metabolism markers respond to MC fluctuations. Moreover, most studies did not report whether participants engaged in any physical activity in the 24–48 h prior to blood sampling.

Finally, one of the most critical limitations in this field is the small sample sizes used in many studies. Given the high biological variability in hormonal responses and bone metabolism, small sample sizes may lack the statistical power necessary to detect meaningful differences between menstrual phases. This could explain why some studies report significant variations while others do not.

Therefore, future research should aim to standardize menstrual phase determination and verification methods, ensure hormonal validation of cycle phases, and include larger, well-controlled sample sizes to improve the reliability and reproducibility of findings.

### Practical Applications

The practical implications of the present findings should be interpreted with caution in a clinical context. In routine practice, bone (re)modelling markers are most commonly assessed in postmenopausal females, and a substantial proportion of premenopausal females use oral hormonal contraception, which further limits the real-world applicability of MC–specific interpretations. In addition, the available evidence does not converge on a single optimal sampling window across the MC, and the logistical challenges of scheduling blood sampling at precise cycle phases reduce the feasibility of translating these findings into actionable clinical recommendations. Importantly, any potential MC–related effects on bone metabolism markers appear modest when compared with other major sources of pre-analytical variability, such as fasting status, time of day, and sample type or handling, which are likely to dominate marker interpretation in routine care.

From a research perspective, however, these findings are highly relevant. Accounting for MC phase, phase verification, and sources of biological variability may help optimize study design, reduce unexplained variance, and improve the interpretability and reproducibility of bone metabolism research conducted under controlled experimental conditions in premenopausal women.

## Conclusion

This review indicates that no consistent pattern of fluctuation in bone metabolism markers across the MC has been established. Although some studies report statistically significant changes in specific markers, findings are inconsistent and characterized by substantial methodological heterogeneity. These results suggest that MC-related variations in bone (re)modelling markers are not uniform and, when present, are likely modest in magnitude and influenced by multiple physiological and methodological factors.

Rather than providing definitive guidance for clinical practice, this review clarifies key sources of divergence across studies, including differences in cycle-phase definition and verification, biomarker selection, and sampling protocols. Furthermore, although sex hormones such as oestradiol and progesterone exert clear regulatory effects on bone cells in vitro, physiological hormonal fluctuations across the MC do not appear to translate into predictable or robust changes in circulating bone metabolism markers in vivo.

## Supplementary Information

Below is the link to the electronic supplementary material.

Supplementary Material 1

Supplementary Material 2

Supplementary Material 3

## Data Availability

The data within this review are secondary data and available through the relevant articles referenced throughout.

## References

[1] Hart NH, Nimphius S, Rantalainen T, Ireland A, Siafarikas A, Newton RU (2017) Mechanical basis of bone strength: influence of bone material, bone structure and muscle action. J Musculoskelet Neuronal Interact 17:114–139 28860414 PMC5601257

[2] Khosla S, Oursler MJ, Monroe DG (2012) Estrogen and the skeleton. Trends Endocrinol Metab 23:576–581 22595550 10.1016/j.tem.2012.03.008 PMC3424385

[3] Khosla S, Monroe DG (2018) Regulation of bone metabolism by sex steroids. Cold Spring Harb Perspect Med 8:a031211 28710257 10.1101/cshperspect.a031211 PMC5749141

[4] Galea GL, Meakin LB, Sugiyama T, Zebda N, Sunters A, Taipaleenmaki H, Stein GS, Van Wijnen AJ, Lanyon LE, Price JS (2013) Estrogen receptor α mediates proliferation of osteoblastic cells stimulated by Estrogen and mechanical Strain, but their acute Down-regulation of the Wnt antagonist Sost is mediated by Estrogen receptor β. J Biol Chem 288:9035–9048 23362266 10.1074/jbc.M112.405456 PMC3610976

[5] Bord S, Ireland DC, Beavan SR, Compston JE (2003) The effects of Estrogen on osteoprotegerin, RANKL, and Estrogen receptor expression in human osteoblasts. Bone 32:136–141 12633785 10.1016/s8756-3282(02)00953-5

[6] Seifert-Klauss V, Prior JC (2010) Progesterone and bone: actions promoting bone health in women. J Osteoporos 2010:1–18 10.4061/2010/845180 PMC2968416 21052538

[7] Gass ML, Kagan R, Kohles JD, Martens MG (2008) Bone turnover marker profile in relation to the menstrual cycle of premenopausal healthy women. Menopause 15:667–675 18327152 10.1097/gme.0b013e31815f8917

[8] Mozzanega B, Gizzo S, Bernardi D, Salmaso L, Patrelli TS, Mioni R, Finos L, Nardelli GB (2013) Cyclic variations of bone resorption mediators and markers in the different phases of the menstrual cycle. J Bone Min Metab 31:461–467 10.1007/s00774-013-0430-4 23479185

[9] Guisado-Cuadrado I, Romero-Parra N, Elliott-Sale KJ, Sale C, Díaz ÁE, Peinado AB (2024) Influence of menstrual cycle and oral contraceptive phases on bone (re)modelling markers in response to interval running. Calcif Tissue Int 115:382–392 39066926 10.1007/s00223-024-01259-4 PMC11405431

[10] Martin D, Cooper SB, Tang JCY, Fraser WD, Sale C, Elliott-Sale KJ (2021) Bone metabolic marker concentrations across the menstrual cycle and phases of combined oral contraceptive use. Bone 145:115864 33508495 10.1016/j.bone.2021.115864

[11] Guzman A, Kurgan N, Moniz SC, McCarthy SF, Sale C, Logan-Sprenger H, Elliott-Sale KJ, Hazell TJ, Klentrou P (2022) Menstrual cycle related fluctuations in Circulating markers of bone metabolism at rest and in response to running in eumenorrheic females. Calcif Tissue Int 111:124–136 35429247 10.1007/s00223-022-00970-4

[12] Hunter SK, Angadi S, Bhargava S, Harper A, Hirschberg J, Levine ALD, Moreau BL, Nokoff KJ, Stachenfeld N, Bermon NS (2023) The Biological basis of sex differences in athletic performance: consensus statement for the American college of sports medicine. Med Sci Sports Exerc 55:2328–2360 37772882 10.1249/MSS.0000000000003300

[13] Elliott-Sale KJ, Minahan CL, De Jonge XAKJ, Ackerman KE, Sipilä S, Constantini NW, Lebrun CM, Hackney AC (2021) Methodological considerations for studies in sport and exercise science with women as participants: A working guide for standards of practice for research on women. Sports Med 51:843–861 33725341 10.1007/s40279-021-01435-8 PMC8053180

[14] Filella X, Guañabens N (2024) Clinical use of bone markers: a challenge to variability. Adv Lab Med / Av En Med De Laboratorio 5:7–14 10.1515/almed-2023-0092 PMC11019881 38634081

[15] Szulc P, Naylor K, Hoyle NR, Eastell R, Leary ET (2017) Use of CTX-I and PINP as bone turnover markers: National bone health alliance recommendations to standardize sample handling and patient Preparation to reduce pre-analytical variability. Osteoporos Int 28:2541–2556 28631236 10.1007/s00198-017-4082-4

[16] Elliott-Sale KJ, Altini M, Doyle-Baker P et al (2025) Why we must stop assuming and estimating menstrual cycle phases in laboratory and field-based sport related research. Sports Med 55:1339 40085421 10.1007/s40279-025-02189-3 PMC12152053

[17] Guisado-Cuadrado I, Romero-Parra N, Cupeiro R, Elliott-Sale KJ, Sale C, Peinado AB (2024) Effect of eccentric-based resistance exercise on bone (re)modelling markers across the menstrual cycle and oral contraceptive cycle. Eur J Appl Physiol 125:1463–1473 39738864 10.1007/s00421-024-05693-y

[18] Lee S, Kumagai T, Hashimoto J, Satoh A, Suzuki T, Yamai K, Ohta S (2012) A change of osteocalcin (OC) and tartrate resistant acid phosphatase 5b (TRACP-5b) with the menstrual cycle. Horm Metab Res 44:699–703 22517558 10.1055/s-0032-1311606

[19] Pitkin RM, Reynolds WA, Williams GA, Hargis GK (1978) Calcium-regulating hormones during the menstrual cycle. J Clin Endocrinol Metab 47:626–632 263315 10.1210/jcem-47-3-626

[20] Chiu KM, Arnaud CD, Ju J, Mayes D, Bacchetti P, Weitz S, Keller ET (2000) Correlation of estradiol, parathyroid hormone, interleukin-6, and soluble interleukin-6 receptor during the normal menstrual cycle. Bone 26:79–85 10617160 10.1016/s8756-3282(99)00243-4

[21] Nielsen HK, Brixen K, Bouillon R, Mosekilde L (1990) Changes in biochemical markers of osteoblastic activity during the menstrual cycle. J Clin Endocrinol Metab 70:1431–1437 2110577 10.1210/jcem-70-5-1431

[22] Gorai I, Taguchi Y, Chaki O, Kikuchi R, Nakayama M, Yang BC, Yokota S, Minaguchi H (1998) Serum soluble interleukin-6 receptor and biochemical markers of bone metabolism show significant variations during the menstrual cycle. J Clin Endocrinol Metab 83:326–332 9467535 10.1210/jcem.83.2.4584

[23] Iida T, Chikamura C, Ishikawa H, Aoi S, Ikeda H, Harada T, Katada K, Ishizaki F, Yatsuya H, Ono Y (2012) Factors predicting bone mineral density (BMD) changes in young women over a one-year study:changes in body weight and bone metabolic markers during the menstrual cycle and their effects on BMD. Acta Med Okayama 66:307–315 22918203 10.18926/AMO/48670

[24] Massafra C, De Felice C, Agnusdei DP, Gioia D, Bagnoli F (1999) Androgens and osteocalcin during the menstrual cycle. J Clin Endocrinol Metab 84:971–974 10084581 10.1210/jcem.84.3.5512

[25] Buchanan JR, Santen RJ, Cavaliere A, Cauffman SW, Greer RB, Demers LM (1986) Interaction between parathyroid hormone and endogenous Estrogen in normal women. Metabolism 35:489–494 3754923 10.1016/0026-0495(86)90003-x

[26] Jurimae J, Vaiksaar S, Maestu J, Purge P, Jurimae T (2011) Adiponectin and bone metabolism markers in female rowers: eumenorrheic and oral contraceptive users. J Endocrinol Invest 34:835–839 21169728 10.3275/7415

[27] Shimizu M, Onoe Y, Mikumo M, Miyabara Y, Kuroda T, Yoshikata R, Ishitani K, Okano H, Ohta H (2009) Variations in Circulating osteoprotegerin and soluble RANKL during diurnal and menstrual cycles in young women. Horm Res 71:285–289 19339793 10.1159/000208802

[28] Schlemmer A, Hassager C, Risteli J, Risteli L, Jensen SB, Christiansen C (1993) Possible variation in bone resorption during the normal menstrual cycle. Acta Endocrinol (Copenh) 129:388–392 8279219 10.1530/acta.0.1290388

[29] Zittermann A, Schwarz I, Scheld K, Sudhop T, Berthold HK, von Bergmann K, van der Ven H, Stehle P (2000) Physiologic fluctuations of serum estradiol levels influence biochemical markers of bone resorption in young women. J Clin Endocrinol Metab 85:95–101 10634371 10.1210/jcem.85.1.6250

[30] Plotkin LI, Bivi N (2014) Chap. 3 - Local regulation of bone cell function. In: Burr DB, Allen MR (eds) Basic and applied bone biology. Academic, San Diego, pp 47–73

[31] Greenberg C, Kukreja SC, Bowser EN, Hargis GK, Henderson WJ, Williams GA (1987) Parathyroid hormone secretion: effect of estradiol and progesterone. Metabolism 36:151–154 3807787 10.1016/0026-0495(87)90009-6

[32] Liu B-Y, Wu P-W, Bringhurst FR, Wang J-T (2002) Estrogen Inhibition of PTH-Stimulated osteoclast formation and attachment in vitro: involvement of both PKA and PKC. Endocrinology 143:627–635 11796519 10.1210/endo.143.2.8614

[33] Nasu M, Sugimoto T, Kaji H, Chihara K (2000) Estrogen modulates osteoblast proliferation and function regulated by parathyroid hormone in osteoblastic SaOS-2 cells: role of insulin-like growth factor (IGF)-I and IGF-binding protein-5. J Endocrinol 167:305–313 11054645 10.1677/joe.0.1670305

[34] Mizokami A, Kawakubo-Yasukochi T, Hirata M (2017) Osteocalcin and its endocrine functions. Biochem Pharmacol 132:1–8 28189726 10.1016/j.bcp.2017.02.001

[35] Wang H, Wang J (2013) Estrogen-related receptor alpha interacts cooperatively with peroxisome proliferator-activated receptor-gamma coactivator-1alpha to regulate osteocalcin gene expression. Cell Biol Int 37:1259–1265 23788330 10.1002/cbin.10148

[36] Nizet A, Cavalier E, Stenvinkel P, Haarhaus M, Magnusson P (2020) Bone alkaline phosphatase: an important biomarker in chronic kidney disease – mineral and bone disorder. Clin Chim Acta 501:198–206 31734146 10.1016/j.cca.2019.11.012

