# Supplementary material for: Understanding Bone Metabolism Biomarker Variability Across the Menstrual Cycle: A Systematic Review
Source: Calcif Tissue Int. 2026 Feb 9;117(1):23. doi: 10.1007/s00223-026-01482-1 (PMC12886325; doi:10.1007/s00223-026-01482-1)
Supplement: Supplementary file 2 — Supplementary Material 2 [file 223_2026_1482_MOESM2_ESM.docx]

**Database Search Details**

PubMed

Search query: (("menstrual cycle"[Title/Abstract] OR "menstrual phase"[Title/Abstract] OR "follicular phase"[Title/Abstract] OR "luteal phase"[Title/Abstract]) AND ("bone remodelling*"[Title/Abstract] OR "bone (re)modelling*"[Title/Abstract] OR "bone metaboli*"[Title/Abstract] OR "bone resorption"[Title/Abstract] OR "bone formation"[Title/Abstract] OR "bone markers"[Title/Abstract] OR "bone turnover"[Title/Abstract])) NOT ("postmenopausal"[Title/Abstract] OR "transgender"[Title/Abstract] OR "cancer"[Title/Abstract] OR "syndrome"[Title/Abstract] OR "endometriosis"[Title/Abstract] OR "mice"[Title/Abstract] OR "rats"[Title/Abstract] OR "animal"[Title/Abstract]).

Results: 76 articles.

Web of Science

Search query: (((((TI=("menstrual cycle" OR "menstrual phase" OR “follicular phase” OR “luteal phase”)) AND AB=("menstrual cycle" OR "menstrual phase" OR “follicular phase” OR “luteal phase”)) AND TI=("bone remodelling*" OR "bone (re)modelling*" OR "bone metaboli*" OR "bone resorption" OR "bone formation" OR "bone markers" OR "bone turnover")) AND AB=("bone remodelling*" OR "bone (re)modelling*" OR "bone metaboli*" OR "bone resorption" OR "bone formation" OR "bone markers" OR "bone turnover")) NOT TI=(“postmenopausal” OR “transgender” OR “cancer” OR “syndrome” OR “endometriosis” OR “mice” OR “rats” OR “animal”)) NOT AB=(“postmenopausal” OR “transgender” OR “cancer” OR “syndrome” OR “endometriosis” OR “mice” OR “rats” OR “animal”).

Results: 18 articles.
