# Supplementary material for: Understanding Bone Metabolism Biomarker Variability Across the Menstrual Cycle: A Systematic Review
Source: Calcif Tissue Int. 2026 Feb 9;117(1):23. doi: 10.1007/s00223-026-01482-1 (PMC12886325; doi:10.1007/s00223-026-01482-1)
Supplement: Supplementary file 3 — Supplementary Material 3 [file 223_2026_1482_MOESM3_ESM.docx]

|  | Buchanan et al. (1986) | Chiu et al. (2000) | Gass et al. (2008) | Gorai et al. (1998) | Guisado‑Cuadrado et al. (2024) (b) | Guisado‑Cuadrado et al. (2024) (a) | Guzman et al. (2022) | Iida et al. (2012) | Jürimäe et al. (2011) | Lee et al. (2012) | Martin et al. (2021) | Massafra et al. (1999) | Mozzanega et al. (2013) | Nielsen et al. (1990) | Pitkin et al. (1978) | Schlemmer et al. (1993) | Shimizu et al. (2009) | Zittermann et al. (2000) |
| --- | --- | --- | --- | --- | --- | --- | --- | --- | --- | --- | --- | --- | --- | --- | --- | --- | --- | --- |
| 1.Aims stated clearly | Yes | Yes | Yes | Yes | Yes | Yes | Yes | Yes | Yes | Yes | Yes | Yes | Yes | Yes | Yes | Yes | Yes | Yes |
| 2. Population clearly specified | Yes | Yes | Yes | Yes | Yes | Yes | Yes | Yes | Yes | Yes | Yes | Yes | Yes | Yes | Yes | Yes | Yes | Yes |
| 3. >50% participation of eligible | No | No | No | No | No | No | No | No | No | No | Yes | No | No | No | No | No | No | No |
| 4. Inclusion/exclusion criteria | Yes | Yes | Yes | Yes | Yes | Yes | Yes | Yes | Yes | Yes | Yes | Yes | Yes | Yes | Yes | Yes | Yes | Yes |
| 5. Sample size power analysis | No | No | Yes | No | No | No | No | No | No | No | No | No | No | No | No | No | No | No |
| 6. Hormones measured prior | Yes | Yes | Yes | Yes | Yes | Yes | Yes | No | No | Yes | No | Yes | No | Yes | No | Yes | No | Yes |
| 7. Sufficient number time points | Yes | Yes | Yes | Yes | Yes | Yes | Yes | Yes | Yes | Yes | Yes | Yes | Yes | Yes | Yes | Yes | No | Yes |
| 8. Different levels of hormone exposure | Yes | Yes | Yes | NR | Yes | Yes | Yes | Yes | Yes | Yes | NR | Yes | Yes | Yes | NR | Yes | NR | Yes |
| 9. Hormone measures valid and reliable | Yes | Yes | NR | Yes | Yes | Yes | Yes | No | Yes | Yes | Yes | Yes | Yes | Yes | Yes | Yes | Yes | Yes |
| 10. Measured duplicated across two cycles | No | No | No | No | No | No | No | No | No | No | No | No | No | No | No | No | No | No |
| 11. Bone metabolism measures valid and reliable | Yes | Yes | NR | Yes | Yes | Yes | Yes | No | Yes | Yes | Yes | Yes | Yes | Yes | Yes | Yes | Yes | Yes |
| 12. Assessors blinded to phase | No | No | No | No | No | No | No | No | No | No | No | No | No | No | No | No | No | Yes |
| 13. Loss of participants <20% | NR | No | Yes | Yes | NR | NR | Yes | Yes | Yes | NR | No | NR | NR | NR | NR | NR | NR | NR |
| 14. Confounding factors adequately controlled | No | No | Yes | Yes | Yes | Yes | Yes | Yes | Yes | Yes | Yes | Yes | Yes | Yes | Yes | Yes | No | Yes |
| Total “Yes” per study | **8** | **8** | **9** | **9** | **9** | **9** | **10** | **7** | **9** | **9** | **8** | **9** | **8** | **9** | **7** | **9** | **5** | **10** |

**Supplementary table 2.** Individual scores for each study using the National Heart, Lung, and Blood Institute Quality Assessment Tool for Observational Cohort and Cross-Sectional Studies.NR, Not Reported
